# Neutrophil-to-Lymphocyte Ratio and Platelet-to-Lymphocyte Ratio in Antenatal Women With Pre-eclampsia: A Case-Control Study

**DOI:** 10.7759/cureus.40338

**Published:** 2023-06-12

**Authors:** Dipanjali Thombare, Anuja Bhalerao, Prachi Dixit, Sulabha Joshi, Priyanka Dapkekar

**Affiliations:** 1 Department of Obstetrics and Gynaecology, NKP Salve Institute of Medical Sciences and Research Centre, Nagpur, IND

**Keywords:** hypertensive disorders, antenatal care, platelet-to-lymphocyte ratio, neutrophil-to-lymphocyte ratio, pre-eclampsia

## Abstract

Background

Pre-eclampsia (PE) is a disorder characterized by hypertension that may occur in a pregnant woman who was normotensive earlier. One of the major factors responsible consists of the inflammatory system being activated with cytokines and chemokines. The normal range of neutrophil-to-lymphocyte ratio (NLR) and platelet-to-lymphocyte ratio (PLR) varies from 1 to 3 and 90 to 210, respectively. Therefore, this study was conducted to compare PE patients with normotensive pregnant women in relation to their NLR as well as PLR.

Methodology

From January 1, 2021, to December 31, 2022, a case-control study was conducted in the department of obstetrics and gynecology at a tertiary care center. Based on the inclusion and exclusion criteria, a total of 140 antenatal women were included and divided into a case group consisting of 70 women with PE and a control group involving 70 normotensive pregnant women. A blood sample for complete blood count testing was collected to determine NLR and PLR.

Results

The mean NLR in the case group and control group was 3.52 ± 1.05 and 3.22 ± 0.88, respectively, with statistically significant results. Additionally, the PLR in the case group was 98.08 ± 18.27, and in the control group, it was 85.25 ± 12.36, having a significant difference between both the groups along with a significant difference in the case group among the severe and non-severe PE.

Conclusion

In antenatal women, NLR and PLR increase with PE along with an increase in NLR and PLR. Additionally, with the availability of a complete blood count, detecting PE ability markers such as the NLR and PLR will be a significant advantage for managing PE to prevent adverse outcomes.

## Introduction

The high rate of maternal mortality and morbidity in developing countries is mainly due to the popular triad consisting of hypertension, hemorrhage, and sepsis, in which the major factor is hypertension. Pre-eclampsia (PE) disorder involves various systems that are characterized by hypertension of 140/90 mm of Hg or higher during pregnancy, which occurs with or without proteinuria after the completion of the 20th week of pregnancy in a pregnant woman who was normotensive before [1,2] and is claimed to affect 8-10% of pregnant women in India. PE is classified into four categories consisting of early onset, which occurs with delivery at <34+0 week’s gestation, preterm PE, which occurs with delivery at <37+0 week’s gestation, late-onset PE, which occurs with delivery at ≥34+0 week’s gestation, and term PE, with delivery at ≥37+0 week’s gestation [3]. The potential causes of PE include hereditary predisposition, abnormal placentation, and immune response [4], and one of the major factors responsible consists of the inflammatory system being activated with cytokines and chemokines [4,5].

According to previous studies, leucocyte activation is crucial in the progression of PE disease [6]. Vascular damage is thought to be caused by a combination of activated leucocytes, platelets, and vascular endothelium [7], and numerous soluble and adhesion molecules are released when platelets are activated, causing interactions between platelets, leucocytes, and endothelial cells [8]. Pathogenesis also involves platelet activation significantly and it is also believed that neutrophil activation is a key factor for the excessive inflammatory response in the maternal vascular system [9].

The oxidative stress indicators, such as total antioxidant ability (TAC), malondialdehyde (MDA), and oxidative stress index (OSI), are more accurate in predicting PE; they are not routinely assessed and are difficult to access, especially in remote places [10,11]. Almost all local and regional medical facilities and laboratories offer complete blood cell count (CBC) testing, which includes differential leucocyte and platelet counts [12]. The normal range of neutrophil-to-lymphocyte ratio (NLR) and platelet-to-lymphocyte ratio (PLR) varies from 1 to 3 and 90 to 210, respectively [13]. Clinical staff and medical professionals in rural areas will have timely and easy access to PE ability markers such as the NLR and PLR, for which the availability of CBC measurement facilities in small places will be a significant advantage for managing PE as soon as possible and preventing its fatal effects.

Numerous studies were carried out for analyzing modifications in NLR and PLR in healthy pregnant women or PE women. However, results from previous studies vary due to different research designs, sample sizes, and fundamental characteristics of the study subjects. Some studies reported higher NLR and PLR in PE whereas some demonstrated no change [14]. Few studies found that change in NLR and PLR is related to the severity of hypertensive disorders of pregnancy [10]. Therefore, this study was conducted to compare PE patients with normotensive pregnant women in relation to their NLR as well as PLR.

## Materials and methods

A case-control study was carried out in the department of obstetrics and gynecology at a tertiary care center from January 1, 2021, to December 31, 2022, after approval from the Institutional Ethical Committee (IEC) with IEC reference number 95/2021. The inclusion criteria consisted of a singleton pregnancy, the case group involving patients admitted to the obstetric ward with PE, and the control group involving healthy normotensive pregnant women attending the outpatient department (OPD) or admitted in the obstetric ward fulfilling the required age criteria as well as the gestational age. Patients having gestational hypertension, chronic hypertension, gestational diabetes mellitus, thyroid disorders, tuberculosis, acquired infections during the early stage of pregnancy, pre-existing vascular diseases or malignancy, history of alcohol or tobacco use, premature rupture of membranes, and history of COVID-19 infection were excluded from the study. Based on the inclusion and exclusion criteria, 140 patients were enrolled in the study by convenient sampling method after receiving written informed consent. The sample size was calculated based on the previous prevalence rates and out of 140 patients, 70 were in the case group involving PE patients and the other 70 were in the control group consisting of normotensive pregnant women.

For cases and control groups, detailed history was obtained consisting of sociodemographic, medical, surgical, physical, menstrual, and obstetric parameters along with the examination. The physical examination involved per abdomen examination, and measuring of blood pressure by using a sphygmomanometer, following which based on the American College of Obstetricians and Gynecologists (ACOG) criteria, patients of PE were categorized as severe and non-severe PE patients in the case group. Obstetric outcomes in the form of gestational age at delivery, mode of delivery, indication in case of cesarean delivery, antepartum, intrapartum, and postpartum complications, and neonatal outcomes were noted. For clinical investigation, 5 ml of venous blood was collected in a clean and dry ethylenediaminetetraacetic acid (EDTA) bulb for CBC test of all the patients, which was measured by a five-part cell counter autoanalyzer in which absolute count of neutrophils, lymphocytes, and platelets was noted. Following this, NLR and PLR were calculated and data were entered into an Excel sheet (Microsoft Corporation, Redmond, WA) for comparison between the two groups.

STATA version 10.1 (StataCorp LLC, College Station, TX) was used to code and analyze the data, and quantitative variables were computed using descriptive statistics. To describe categorical variance, frequency and percentages were utilized. Inferential statistics include a test of significance and p-value for comparison of parameters between the case group (PE) and the control group. The chi-square test compared attributes in two groups while the t-test compared the mean. For all comparisons, a p-value of 0.05 or lower was considered statistically significant.

## Results

A total of 140 women were enrolled in the study, out of which 70 were in the case group (PE) and 70 were in the control group (normotensive). Out of 70 women in the PE group, 35 had severe PE and the other 35 had non-severe PE. All the patients in the normotensive group were followed till delivery and none of them developed PE. Age-wise distribution of women in both groups is demonstrated in Table 1.

**Table 1:** Distribution of women according to age PE: pre-eclampsia.

Age group (years)	PE group (n = 70)		Normotensive group (n = 70)	
	No	%	No	%
20-24	32	47	28	40
25-29	29	42	30	42.8
30-35	09	11	12	17.2
Total	70	100	70	100
Pearson- chi^2^ = 0.712; p-value = 0.7004				
Mean ± SD	25.92 ± 3.73		25.57 ± 4.05	

The distribution of women according to the locality is demonstrated in Table 2.

**Table 2:** Distribution of women according to the locality PE: pre-eclampsia.

Locality	PE group (n = 70)		Normotensive group (n = 70)	
	No	%	No	%
Rural	58	82.8	54	77.1
Urban	12	17.2	16	22.9
Total	70	100	70	100
Pearson chi^2^ = 0.7143; p-value = 0.39				

The distribution of women according to socioeconomic status is demonstrated in Table 3.

**Table 3:** Distribution of women according to the socioeconomic status PE: pre-eclampsia.

Socioeconomic status	PE group (n = 70)	Normotensive group (n = 70)
	n (%)	n (%)
Upper	0 (0)	0 (0)
Upper middle	31 (44.2)	34 (48.5)
Lower middle	16 (22.8)	18 (26.0)
Upper lower	23 (33.0)	18 (25.5)
Lower	0 (0)	0 (0)
Pearson chi^2^ = 0.25; p-value= 0.305		

The distribution of women according to the antenatal care (ANC) booking status is demonstrated in Table 4.

**Table 4:** Distribution of women according to the ANC booking status ANC: antenatal care; PE: pre-eclampsia.

Booking type	PE group (n = 70)		Normotensive group (n = 70)	
	No	%	No	%
Booked	58	82.8	62	88.5
Unbooked	12	17.2	08	11.5
Total	70	100	70	100
Pearson chi^2 ^= 0.993; p-value = 0.16				

The distribution of women by gestational age demonstrated that the majority of women involving 71.5% each in both the groups were between 37+1 to 40 weeks of gestation, as shown in Table 5.

**Table 5:** Distribution of women according to gestational age in weeks PE: pre-eclampsia.

Gestational age (weeks)	PE group (n = 70), number (%)	Normotensive group (n=70), number (%)	
34+1-37	17 (24.2)	12 (17.1)	
37+1-40	50 (71.5)	50 (71.5)	
40+1-42	03 (04.3)	08 (11.4)	
Pearson chi-square= 3.13	p-value = 0.2		

The distribution of women according to the obstetric history showed maximum women were primigravida in the PE group and multigravida in the normotensive group, as demonstrated in Table 6.

**Table 6:** Distribution of women according to obstetric history PE: pre-eclampsia.

Obstetrics history	PE group (n = 70), number (%)	Normotensive group (n = 70), number (%)
Primigravida	37 (52.8)	32 (45.7)
Multigravida	33 (47.2)	38 (54.3)
Nulliparous	15 (21.4)	18 (25.8)
Para1	12 (17.2)	13 (18.5)
Para2	6 (08.6)	07 (10.0)

The distribution of women according to body mass index (BMI) is demonstrated in Table 7.

**Table 7:** Distribution of women according to body mass index PE: pre-eclampsia; BMI: body mass index.

BMI	PE group (n = 70), number (%)	Normotensive group (n = 70), number (%)
<18.5 (underweight)	0	0
18.5-<25 (normal)	53 (75.7%)	48 (68.6%)
25-<30 (overweight)	13 (18.6%)	15 (21.4%)
≥30 (obese)	4 (5.7%)	7 (10%)
Mean ± SD	23.86 ± 3.58	23.73 ± 3.43
Chi-square = 1.209; p-value = 0.546		

The relationship between the severity of PE and NLR with statistically significant results is demonstrated in Table 8.

**Table 8:** Relation between pre-eclampsia and NLR PE: pre-eclampsia; NLR: neutrophil-to-lymphocyte ratio.

NLR	PE group (n = 70)			Normotensive group (n = 70)
	Severe PE	Non-severe PE	Total	
≤3	4	26	30	55
>3	31	09	40	15
Total	35	35	70	70
Chi-square =18.72; p-value = <0.001				

The relationship between the severity of PE and NLR with statistically significant results is demonstrated in Table 9.

**Table 9:** Relationship between the severity of pre-eclampsia and PLR PE: pre-eclampsia; PLR: platelet-to-lymphocyte ratio.

PLR	PE group (n = 70)			Normotensive group (n = 70)
	Severe PE	Non-severe PE	Total	
≤90	7	21	28	60
>90	28	14	42	10
Total	35	35	70	70
Chi-square = 28; p-value = <0.001				

The relationship between the mode of delivery consisting of lower segment cesarean section (LSCS) and vaginal delivery (VD) and the severity of PE in NLR and PLR with statistically significant results is demonstrated in Table 10.

**Table 10:** Relationship between mode of delivery and severity of pre-eclampsia in NLR and PLR NLR: neutrophil-to-lymphocyte ratio; PLR: platelet-to-lymphocyte ratio; VD: vaginal delivery; LSCS: lower segment cesarean section.

Mode of delivery	PE group (n = 70)			Normotensive group (n = 70)
	Severe PE	Non-severe PE	Total	
VD	9 (12.85%)	17 (24.28%)	26	48 (68.5)
LSCS	26 (37.16%)	18 (25.71%)	44	22 (31.5)
Chi-square = 13.87; p-value = <0.001				

## Discussion

The present study was conducted with 140 antenatal women to compare PE patients with normotensive pregnant women in relation to their NLR as well as PLR. The case group (PE) consisted of 70 women, out of which 35 had severe PE and the other 35 had non-severe PE. The control group consisted of normotensive pregnant women. The mean age of patients in the case group was 25.92 ± 3.73 years whereas the mean age of patients in the normotensive group was 25.57 ± 4.05 years. Similarly, in a study done by Cadden et al., the mean age in the case and control group was 24.6 ± 1.7 years and 22.6 ± 0.7 years, respectively, which was lower than the present study [15], whereas Canzoneri et al. reported that the mean age was 23.3 ± 5.4 years and 23.5 ± 6 years, respectively [8]. In contrast to the above studies, Toptas et al. reported a mean age of 29.4 ± 6.1 years and 26.8 ± 6.4 years, which was higher in comparison to the present study [10].

In the present study, the majority of women (67%) in the case group and control group (74.5%) were from the middle class, which correlates with the study by Sachan et al. in which the majority of women in the case group (67.6%) and the control group (64.7%) were from middle class [10]. The mean gestational age of women in the present study was 37.66 ± 1.57 weeks for the PE group and 38.08 ± 1 weeks for the normotensive group. Toptas et al. reported that the mean gestational age of women in PE and the normotensive group was 36.8 ± 2.0 weeks and 37.2 ± 2 weeks, respectively [10]. Additionally, Amidu et al. concluded a mean gestational age of 37.3 ± 0.66 weeks and 39.0 ± 1.6 weeks, respectively [16]. Furthermore, Bozdağ et al. reported a mean gestational age of 36 in the case group and 39 in the control group. In contrast to the above studies, the present study demonstrated statistically insignificant results [17].

In the present study, the mean BMI in PE and the normotensive group was 23.86 ± 3.58 kg/m2 and 23.73 ± 3.43 kg/m2, respectively, demonstrating that maximum women were under the normal range. Sachan et al. in their study reported BMI of 25.13 ± 0.96 kg/m2 and 22.10 ± 0.52 kg/m2, respectively, demonstrating maximum women under the normal range [18], whereas Cadden et al. found a mean BMI of 29.7 ± 2.6 kg/m2 and 29.0 ± 3.1 kg/m2, respectively, concluding maximum women were overweight [15].

The distribution of women according to NLR and PLR in the PE and normotensive group in the present study demonstrated that the mean NLR in the PE and normotensive groups was 3.52 ± 1.05 and 3.22 ± 0.88, respectively, with statistically significant results. Additionally, the PLR in the case group was 98.08 ± 18.27, and in the control group, it was 85.25 ± 12.36, having a significant difference between both the groups along with a significant difference in the case group among the severe and non-severe PE. Similarly, Sachan et al. demonstrated that the mean NLR in the case group with non-severe PE was 3.38 ± 0.16, and in severe PE, it was 4.26 ± 0.31. In the normotensive group, the NLR was 3.14 ± 0.16 with statistically significant results [18]. Additionally, Amidu et al. reported that the case group had a mean NLR of 4.84 ± 2.37 and a mean NLR of 12.76 ± 7.48 in the control group with a statistically significant difference between both the groups but the control group had increased NLR, which signifies increased lymphocyte count and decreased neutrophil in advanced PE [16].

In the present study, the majority of women in the case group (63%) underwent LSCS and the remaining 37% underwent vaginal delivery, whereas, in the control group, 31.5% and 68.5% underwent LSCS and vaginal delivery, respectively, with statistically significant results between both groups. Whereas, Canzoneri et al. reported a statistically significant difference between groups in which case group had 71.25% delivered vaginally and 28.75% delivered by LSCS in non-severe PE and 52.5% vaginal delivery and 47.5% LSCS in severe PE. In the control group, 70% demonstrated vaginal delivery. The increase in the rate of LSCS in PE women was due to the need for early termination as required in severe hypertensive disorders of pregnancy [8].

## Conclusions

The present study concludes that NLR and PLR increase in antenatal women with PE along with an increase in NLR and PLR according to the severity of PE. As PE causes fatal complications to both mother and fetus, detection of PE early in pregnancy is necessary. These raised NLR and PLR obtained from CBC can be used for the prediction of PE and also helps in the detection of the severity of PE, which is easily available at all healthcare centers.
